# Immediate effect of lung expansion techniques in neurosurgery patients detected by electrical impedance tomography: a randomized crossover study

**DOI:** 10.1186/2197-425X-3-S1-A991

**Published:** 2015-10-01

**Authors:** CCA Morais, C Rattes, M Bandeira, L Monte, SL Campos, D Brandão, MBP Amato, AD Andrade

**Affiliations:** Universidade Federal de Pernambuco, Physiotherapy, Recife, Brazil; Universidade de São Paulo, Pulmonary Division, Sao Paulo, Brazil

## Introduction

The survival of patients with lesions in the central nervous system is usually accompanied by physical and mental sequelae. These impairments favor the prolonged restriction to the bed, which may contribute with changes in respiratory function. In this context, lung re-expansion techniques are used to prevent or treat the various respiratory complications.

## Objectives

To compare the effect of Breath Stacking (BS) and Expiratory Positive Airway Pressure (EPAP) techniques on the regional lung ventilation, aeration and on the maintenance of the therapeutic effect of lung expansion in non-cooperative neurosurgery patients with prolonged bed rest. Secondary aims included to evaluate the influence of these techniques on cardiorespiratory system.

## Methods

This was a randomized crossover study. Ten patients were included with Glasgow Coma Scale between 3-10 points, 18-65 years old, undergoing neurosurgery, unable to respond to the command and restricted to bed. The regional lung ventilation and aeration changes were assessed by EIT device (Enligh 1800, Timpel, São Paulo, Brazil). For interventions, EPAP was applied through a Spring Load valve resistor, adjusted with a pressure of 10 cmH2O. BS was performed three times with one minute intervals between them. The volume was stacked and maintained until 40 seconds.

## Results

The regional lung ventilation during the BS was significantly higher in the inter-intervention and intra-intervention analyses when compared the baseline with the first minute (all *P* < 0.001). The regional lung aeration was increased after both techniques significantly (all *P* < 0.001), however, the BS increase more than EPAP (*P* < 0.001). There were no differences in the duration of therapeutic effect between the EPAP (4.6 ± 3.7 minutes) and BS (2.3 ± 2.0 minutes) (*P* = 0.103) and there were no clinically significant differences on cardiorespiratory variables.

## Conclusions

The BS and EPAP techniques promoted significant changes on lung volumes, but not maintained the therapeutic effect of lung expansion for a long time, and did not generate adverse effects on the cardiovascular system in non-cooperative neurosurgery patients with prolonged bed rest.

## Grant Acknowledgment

This work was supported by grants from the Conselho Nacional de Desenvolvimento Científico e Tecnológico, Brazil (CNPq), and the Fundação de Amparo a Ciência e Tecnologia do Estado de Pernambuco (FACEPE).
